# Tailoring Topological Magnetic States in Multilayer Nanostructures: Bloch Points, Chiral Bobbers, and Skyrmion Tubes

**DOI:** 10.3390/nano15191473

**Published:** 2025-09-25

**Authors:** Zukhra Gareeva, Viktoria Filippova, Shamil Gareev, Ildus Sharafullin

**Affiliations:** 1 Institute of Molecule and Crystal Physics, Subdivision of the Ufa Federal Research Centre of the Russian Academy of Sciences, 450075 Ufa, Russia; mukhamadeeva.vika@mail.ru (V.F.);; 2 Institute of Physics and Technology, Ufa University of Science and Technology, 450000 Ufa, Russia

**Keywords:** magnetic nanostructures, Dzyaloshinskii–Moriya interaction, Bloch points, skyrmions

## Abstract

Topological magnetic textures—including skyrmions, Bloch points, and chiral bobbers—exhibit extraordinary properties with significant potential for advanced information technologies. However, achieving precise control over specific topological states requires an understanding of their formation mechanisms and stabilization criteria in nanoscale materials. Our work addresses this challenge by investigating how tailored interactions in ferromagnetic multilayers govern the emergence of specific topological configurations. In this study, we investigate topological magnetic structures in ferromagnetic multilayers, focusing on the interplay between magnetic anisotropy, the Dzyaloshinskii–Moriya interaction, and interlayer exchange coupling. We demonstrate how these interactions govern the formation and stability of diverse 3D topological configurations, including Bloch-point-like structures, conical skyrmions, chiral bobbers, and skyrmion tubes. Optimal conditions for stabilizing specific defect types have been identified and phase diagrams have been constructed as a function of material parameters. These findings provide insights into the controlled design of magnetic textures for advanced spintronic applications.

## 1. Introduction

In recent years, magnetic skyrmions and related topological spin textures have garnered significant interest for their exceptional properties and promising next-generation information technologies. These nanoscale magnetic whirls exhibit remarkable features such as topological protection, ensuring stability against thermal fluctuations and external perturbations, as well as ultra-small size down to nanometers, enabling high-density data storage. Additionally, their low threshold current densities for driven motion make them highly energy-efficient for spintronic applications, while their non-trivial spin dynamics open new avenues for neuromorphic computing and unconventional logic devices [1,2,3,4].

Initially observed in non-centrosymmetric bulk materials with intrinsic Dzyaloshinskii–Moriya interaction (DMI), skyrmions were later stabilized in thin films through interfacial DMI, expanding their design flexibility. This advancement has led to the development of “topotronics,” a rapidly growing field that explores the interplay between spatial topology and novel physical phenomena [5]. Beyond conventional skyrmions, a diverse array of topological spin textures—including merons, antiskyrmions, Bloch points, and hopfions—has been identified, with each offering distinct advantages for different applications [4,6,7,8,9,10].

The mechanisms governing the formation and stability of these topological defects vary considerably between bulk materials and thin films. In bulk crystals, the absence of inversion symmetry naturally induces bulk DMI, favoring chiral spin structures. In contrast, thin-film systems rely on interfacial symmetry breaking to generate DMI, which allows for greater flexibility in tuning magnetic interactions through material engineering. Multilayer heterostructures enabled by advances in epitaxial growth and precise interface control offer several key advantages over single-layer films. By carefully designing the layer-stacking sequence, researchers can enhance DMI and tailor interlayer exchange coupling, creating optimized conditions for spin texture formation [11,12]. Moreover, the integration of layers exhibiting magnetic anisotropy (MA) of “easy axis” type (PMA—perpendicular MA) and MA of the “easy-plane” type (IMA—in-plane MA) introduces spatially varying potential landscapes, enabling the stabilization of complex and metastable topological states that are inaccessible in simpler systems [13,14].

Of particular interest are heterostructures that integrate PMA and IMA layers, as their competing anisotropies can give rise to exotic three-dimensional (3D) spin textures. While two-dimensional (2D) skyrmions have been extensively studied, 3D topological states—such as skyrmion tubes, chiral bobbers, and vertically coupled domain walls—present new opportunities for advanced spintronic applications. These configurations benefit from extended stability regimes, richer dynamical behaviors, and the potential for vertical information transport, making them promising candidates for multi-level memory architectures and neuromorphic computing systems.

The past decade has witnessed remarkable progress in fabricating tailored magnetic heterostructures, enabled by advances in epitaxial growth and interface control. Particularly promising are systems combining layers with PMA and IMA, which introduce spatially varying potential landscapes for spin textures. When interfaced with heavy metals or magnetoelectric materials, these systems gain additional control knobs through interfacial Dzyaloshinskii–Moriya interaction (DMI) and voltage-tunable anisotropy.

In this work, we investigate an exchange-coupled multilayer system that hosts a variety of topological magnetic textures, including Bloch points and skyrmions, and explore the conditions required for their selective stabilization. We consider ferromagnetic film, comprising bi-layers with PMA sandwiched between the layers with IMA; this design allows us to stabilize Bloch-points and concomitant states induced by the external magnetic field applied along the normal to the film surface. Our findings demonstrate how the careful balancing of anisotropy, DMI, and interlayer coupling can create a toolbox for spin textures—from compact chiral bobbers suitable for logic operations to extended skyrmion tubes adjustable for three-dimensional memory devices. By analyzing the influence of magnetic anisotropy, DMI, and interlayer exchange coupling, we provide insights into the controlled design of topological spin structures, paving the way for their integration into future nanomagnetic and spintronic devices.

The manuscript is organized as follows: Section 2 details the methodology; in this Section we describe the models used to analyze the formation and stability of topological magnetic states in magnetic multilayers and materials, in which the described structures can be observed. Section 3 presents the key findings on the stabilization of topological defects, including a detailed analysis of the observed configurations—Bloch points, chiral bobbers, and skyrmions—along with their distinguishing characteristics. Section 4 summarizes the key insights from this study and discusses their implications.

## 2. Methods and Materials

In this section, we describe the methods employed to investigate topological magnetic structures, particularly Bloch points and their derivatives, and materials where such structures can be observed. The methodology combines theoretical modeling with advanced simulation techniques to establish the conditions for topological defect formation and to identify suitable material platforms for experimental realization.

Building upon previous theoretical studies [15,16], we propose a system that supports vortex states with alternating polarities, where exchange-coupled ferromagnetic films serve as a suitable platform. The system consists of multiple ferromagnetic layers with distinct magnetic properties, where variations in magnetic anisotropy and the Dzyaloshinskii–Moriya interaction (DMI) govern the formation of topological states. The multilayer stack features inner layers with perpendicular magnetic anisotropy (PMA, *K*_2,3_ > 0) sandwiched between boundary layers exhibiting in-plane anisotropy (IMA, *K*_1,4_ < 0). This configuration enables selective magnetization switching in the softer layers under external stimuli while maintaining stable magnetization in the harder layers, facilitating the creation and control of complex three-dimensional spin structures.

The system’s energetics are described by a free-energy functional that incorporates key magnetic interactions:

(1) $$F=\sum_{i=1}^{4}(\underset{V}{∭}dVA_{i}{\left(\partial_{\mu }m_{i\alpha }\right)}^{2}+K_{i}{\left(m_{i}\cdot n\right)}^{2}-\frac{1}{2}\mu_{0}M_{s}\left(m_{i}\cdot H_{m}\right)-\mu_{0}M_{s}\left(m_{i}\cdot H\right)-F_{i,interls}+F_{i,DMI})$$

where

(2) $$\begin{array}{l} F_{i,interls}=\iint_{S}dS\;J_{L_{i}L_{i+1}}\left(m_{i}\cdot m_{i+1}\right)\\ F_{i,DMI}=t\iint_{S}dS\;D[\left(m_{ix}\frac{\partial m_{iz}}{\partial x}-m_{iz}\frac{\partial m_{ix}}{\partial x}\right)+\left(m_{iy}\frac{\partial m_{iz}}{\partial y}-m_{iz}\frac{\partial m_{iy}}{\partial y}\right)] \end{array}$$

where *A_i_* is the constant of non-uniform exchange interaction in the *i*-th layer, *α, μ = x, y, z*; *K_i_* is the constant of magnetic anisotropy in the *i*-th layer (*K*_1,4_ < 0, *K*_2,3_ > 0), ***n*** is the normal to the film; ***m**_i_* is the unit magnetization vector in the *i*-th layer; ***H_m_*** is the magnetostatic field and ***H*** is the external magnetic field; here we take ***H*** = (0, 0, *H*), $J_{L_{i}L_{i+1}}$ is the constant of interlayer exchange; *D* is the constant of the DMI, and *t* is the thickness of the chiral layer.

To investigate this system, we implemented a computational approach based on micromagnetic simulations to analyze nanoscale magnetic configurations and their transformations. The simulations were conducted using OOMMF (version 2.0b0) software [17], whose modular architecture allowed us to define the layer-specific material parameters and numerical settings required for the system under consideration. The exchange stiffness constants were layer-dependent, with *A*_1_ = *A*_4_ = 2.9 × 10^−12^ J/m for the top and bottom IMA layers, and *A*_2_ = *A*_3_ = 4.0 × 10^−12^  J/m for the inner PMA layers. Anisotropy constants were defined as *K*_1_ = *K*_4_ = −7 × 10^5^  J/m^3^ for the easy-plane IMA layers, *K*_2_ = 2 × 10^5^  J/m^3^ and we varied *K*_3_ in a range [2 × 10^3^–2×10^5^] J/m^3^ for the easy-axis PMA layers. Interlayer exchange coupling constants were set to *J*_12_* = J*_34_ = 3.5 × 10^−12^  J/m^2^ for the top/bottom interfaces and *J*_23_ = 0.2 × 10^−12^  J/m^2^ for the middle interface. The saturation magnetization was set to *M_s_* = 50  kA/m uniformly across all layers, and a Gilbert damping constant of α = 0.5 was employed to facilitate rapid convergence to equilibrium states by suppressing the precessional dynamics, a standard approach for quasi-static calculations [18]. As experiments have shown, the DMI strengths can vary widely across material classes: reaching values of 1.4–1.8 mJ/m^2^ in the heavy-metal/ferromagnet multilayers [19] like Ir/Fe/Co/Pt or Pt/Co/MgO, while being considerably smaller (on the order of 0.01–0.002 mJ/m^2^) in oxide-based systems such as iron garnet films [20,21] and 2D systems like Janus magnets. We also highlight that emerging approaches, such as voltage-induced charge redistribution at metal–oxide interfaces, offer promising routes to dynamically enhance DMI [22]. In our calculations, the Dzyaloshinskii–Moriya interaction (DMI) strength was varied within the range *D* = 1–9 × 10^−4^  J/m^2^.

Numerical integration of the Landau–Lifshitz–Gilbert equation was performed using the Oxs_RungeKuttaEvolve module, which implements an adaptive fifth-order Runge–Kutta method with automatic timestep control to ensure stability and accuracy. Boundary conditions consisted of natural (free) exchange boundaries, as implemented by Oxs_Exchange6Ngbr, and open (free-space) magnetostatic boundaries via Oxs_Demag. The convergence criteria valid for quasistatic calculations was ∣*d**m***/*dt*∣ < 0.01  deg/ns. The model accounted for standard micromagnetic energy contributions (exchange, Zeeman, uniaxial anisotropy, DMI) within each ferromagnetic (FM) layer. The coupling between adjacent FM layers was mediated by interlayer exchange coupling, implemented using a custom energy term in the Hamiltonian [17]. The system was set in a perpendicularly magnetized state and relaxed under varying field conditions to observe topological transitions. We employed an external magnetic field as the primary stimulus, while noting that similar processes can also be induced by electric currents.

The system dimensions were 200 × 200 × 96 nm^3^ (lateral size *d* = 200 nm, individual layer thickness *t_i_* = 24 nm, total thickness *t* = 96 nm), discretized into cells *a* × *a* × *c* nm^3^. Mesh sizes of *a* = 2, 3, 5 nm, and *c* = 2, 3 nm were tested. The grid size is crucial for convergence, especially for complex 3D states like skyrmions and Bloch points. The chosen base cell dimensions (*a* = 5 nm, *c* = 3 nm) were considerably smaller than the calculated exchange length (12–14 nm), ensuring resolution of relevant magnetization textures. Convergence tests with finer grids confirmed the stability of our key findings. We noted that the continuum micromagnetic model cannot resolve the atomic-scale singularity of a true Bloch point [23]; therefore, we refer to these structures as “Bloch-like points” or “BP-states,” indicating they are micromagnetic analogs.

This initial investigation focuses on Bloch-point type topological defects, characterizing how their nucleation and stability depend on variations in magnetic anisotropy and interlayer exchange coupling. Building on this foundation, we then introduce DMI selectively into specific layers to explore the emergence of novel DMI-stabilized topological states, their stability thresholds relative to key material parameters and transformation pathways between different topological configurations.

In this study we employ rare-earth iron garnet (RIG) films (M_3_Fe_5_O_12_, where M = Y or rare-earth ions) as our primary material platform, selected for their exceptional versatility in hosting and stabilizing topological magnetic states. The garnet crystal structure provides an ideal playground for magnetic property engineering due to its remarkable chemical flexibility. The M-site cation positions readily accommodate various rare-earth substitutions, enabling exceptionally broad and precise tunability of magnetic anisotropy [24]. Although the properties of PMA-IMA garnet bilayers are well-characterized [25], emerging research also reveals DMI in RIG, which is confirmed by the first-principles calculations [21] and experimental observations of interfacial DMI in RIG heterostructures [20]. The demonstrated capability to induce DMI without heavy-metal interfaces suggests new possibilities for designing low-damping topological systems, potentially overcoming some limitations of conventional heavy-metal/ferromagnet heterostructures. This combination of properties makes iron garnets particularly valuable for our comparative study of magnetic anisotropy-stabilized topological defects, DMI-induced chiral magnetic textures and hybrid states emerging from their interplay.

Note that other material systems with comparable parameters may exhibit similar effects to those discussed below. Through manipulation of system parameters—including DMI strength, magnetic anisotropy, interlayer exchange coupling, and geometric confinement—we demonstrate the ability to stabilize a rich variety of topological magnetic structures. In the following sections, we focus specifically on three particularly significant classes of three-dimensional topological defects: Bloch points, chiral bobbers and tube skyrmions.

## 3. Topological Defects and Their Properties

**Figure 1:**
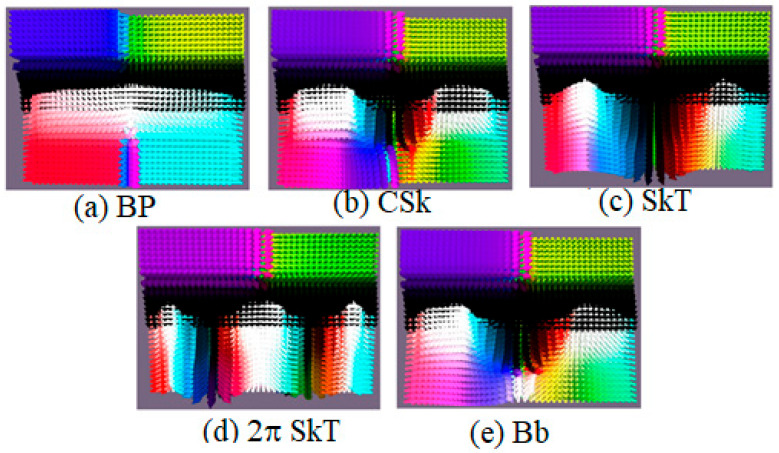
Schematic illustrations and cross-sectional profiles of key three-dimensional topological magnetic states, using the HSL color scale. The panels show the calculated distribution of magnetic moments (represented by arrows) for: (**a**) a Bloch point (BP), (**b**) a conical skyrmion (CSk), (**c**) a skyrmion tube (SkT), (**d**) a chiral bobber (Bb), and (**e**) a 2π-skyrmion tube (2π-SkT).

Let us consider and classify topological defects found in nanoscale magnetic films. The considered structure incorporates inner layers characterized by perpendicular magnetic anisotropy (PMA, “easy axis”), each with tuned anisotropy constants, interleaved between outer boundary layers engineered with in-plane magnetic anisotropy (IMA, “easy plane”). The application of an external magnetic field in the “easy axis” direction triggers magnetization reversal within the soft magnetic layer. In systems lacking the Dzyaloshinskii–Moriya interaction (DMI), this process yields a Bloch point–like (BP) configuration. The introduction of chiral interactions via DMI leads to the emergence of novel three-dimensional topological states, including conical skyrmions (CSk), skyrmion tubes (SkT), and chiral bobbers (Bb), shown in Figure 1.

Each structure exhibits distinct characteristics: BP features a compact, point-like singularity at its core with a region of vertically aligned moments and undefined magnetization at the very center. CSk is characterized by a smooth, Bloch-type rotation of magnetization from the center to the periphery, combined with a vortex-like spin texture in the bottom layer. SkT represents the vertical extension of a skyrmion, maintaining a Bloch-type magnetization rotation from center to edge throughout multiple layers. Bb is distinguished by a Bloch-type rotation in its upper part and a reversed magnetization orientation at the core of the bottom layer, forming a distinct “bobber” shape. Finally, 2π-SkT is a complex state defined by a double (2π) Bloch-type rotation of magnetization from the center to the edges. These configurations are stabilized in the multilayer system under specific conditions of magnetic anisotropy, the Dzyaloshinskii–Moriya interaction, and interlayer coupling, as detailed in the following subsections.

Below, we provide a brief description of the most interesting structures—Bloch points, chiral bobbers, and skyrmion tubes—focusing on the conditions required for their emergence in the structure explored in this work.

### 3.1. Bloch Points in Multilayered Structures

A Bloch point (BP) is a zero-dimensional topological defect [26]. By default, the term refers to a three-dimensional (3D) singularity in a magnetic vector field. The magnetization at a BP’s core is undefined. However, the magnetization distribution in its vicinity can vary, allowing BPs to be classified by polarity, chirality, and vorticity [27,28]. Despite the singularity in magnetization, the energy of a BP remains finite. Assuming simple BP ansatz $m=\frac{r}{r}$ one can show that $F=\iint dVA_{i}{\left(\partial_{\mu }m_{i\alpha }\right)}^{2}=4\pi AR$. The structure and energetics of Bloch points were first studied by Feldtkeller [29], who also coined the term. In magnetic systems with exchange interactions, the considered topological defects emerge as metastable states. The magnetostatic energy contribution to the BP state was evaluated by Döring [30]. During the 1970s–1980s, BPs were proposed as an explanation for certain domain wall phenomena [29,31]. Experimental evidence supported their existence through observations such as Bloch-line displacements and polarity inversion phenomena in thin films featuring non-symmetric Bloch-type domain walls. Key experiments—including pulsed-field Bloch-line dynamics, unconventional domain wall motion in iron garnet and nickel films, and asymmetric wall behavior—provided confirmation of BP configurations [31]. Recent experimental advances have demonstrated that the merging and nucleation of skyrmion tubes can generate Bloch points, accompanied by topological charge transitions from ±1 to 0 [4,32].

It is important to note that the term “singularity” is primarily a mathematical concept. In physical systems, this singularity is accommodated within the interatomic lattice space, with magnetization in the surrounding region oriented in all possible 3D directions. While the continuous model suggests a singularity, the discrete nature of magnetic moments at the atomic scale prevent any physical paradox [4].

Bloch points possess a variety of remarkable properties. Like any singularity, they can trigger a cascade of new topological structures, which is why research on BPs has recently experienced a renaissance. Today, imaging techniques like magnetic force microscopy (MFM) and Lorentz transmission electron microscopy (LTEM) enable direct visualization of BPs [18], while micromagnetic simulations deepen their theoretical understanding [18,33,34]. Ongoing research continues to explore topological defects, including Bloch points, particularly in systems exhibiting a Dzyaloshinskii–Moriya interaction (DMI). At the same time, systems hosting vortex states of different polarities, considered to be potential precursors for Bloch point formation, can also be realized in structures without DMI, where additional research is required.

**Figure 2:**
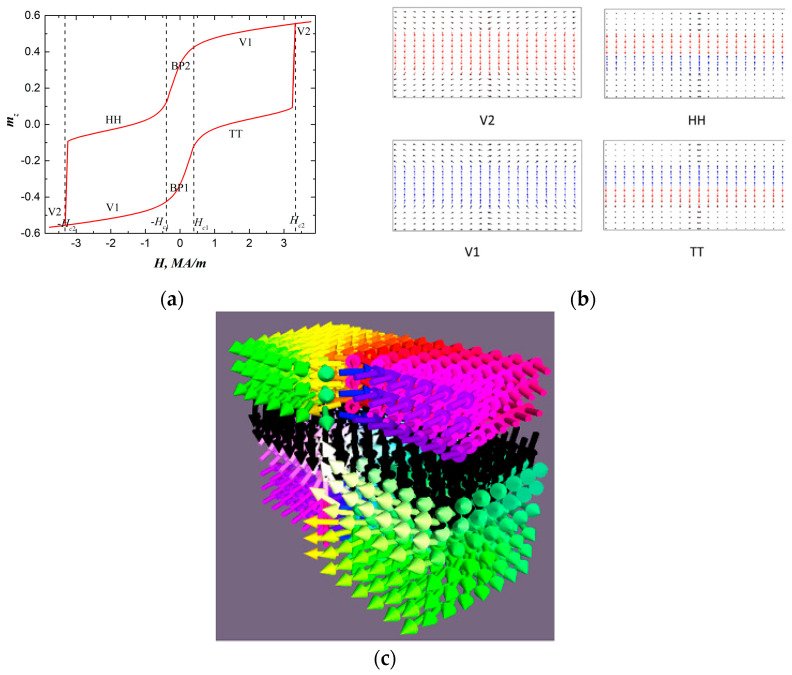
(**a**) Hysteresis loop illustrating magnetization and reversal magnetization processes, symbols V1 and V2 correspond to the states with uniform distribution of magnetic moments in the inner layers along EA direction and vortex-like distribution in the outer layers. HH—“head-to-head” and TT—“tail-to-tail” magnetic moment configurations within the inner layers, symbols BP1 and BP2 correspond to BP-like state with opposite polarities (*p* = +1 for BP1, *p* = −1 for BP2), (**b**) distribution of magnetic moments projected onto *yz*—plane phases, (**c**) distribution of the magnetic moments in Bloch-point-like state at *H* = 0, represented using the HSL color scale.

Concerning the system under consideration, when the Bloch-point-like configurations form during the magnetization process in the cases when DMI is absent or in the cases with chiral layers with a weak DMI, the relevant ratios between *D* and *K* constants are required. At first, we examined systems in the absence of DMI to isolate the fundamental roles of magnetic anisotropy and interlayer exchange coupling. Figure 2a shows hysteresis loop in the system under consideration (*K*_2_ = 2·10^5^ J/m^3^ and *K*_3_ = 2·10^3^ J/m^3^, *D* = 0). The bending of the hysteresis curve signifies the emergence of distinct magnetic phases: phases V_1,2_ with uniform magnetization in the inner layers, domain wall-like structures HH and TT with “head-to head” and “tail-to-tail” orientations of magnetic moments in the inner layers, and BP_1,2_—Bloch-point-like phases distinguished by the polarities of the core (Figure 2b). We also denote the areas of phase’s stability and the critical fields required for transitions between the phases: ±*H_c_*_1_ stands for the transition into Bloch-like state, and ±*H_c_*_2_ stands for the transition into domain-wall like states. HH and TT configurations are stable within the magnetic fields ranging from [±*H_c_*_2_, ±*H_c_*_1_], for given parameters *H_c_*_2_~2580 kA/m, *H*_*c*1_~400 kA/m. The spatial distribution of magnetic moments in the BP configuration and its cross-section along the YZ-plane are visualized in Figure 2c.

The detailed diagrams of magnetic states emerging in the structure during direct magnetization processes are given in Ref. [15]. These findings demonstrate that Bloch-point-like topological defects can be stabilized in the proposed system through precise control of magnetic parameters. This system is particularly suitable for realizing such states due to its multiple adjustable parameters: the anisotropy of all four layers, DMI constant, external magnetic field, and interlayer exchange coupling (both magnitude and sign). Systematic variation in these parameters reveals specific conditions for BP formation.

As demonstrated above, Bloch points form in systems where the boundary layers exhibit identical magnetic anisotropy that significantly exceeds that of inner layers. The configuration shown in Figure 1 corresponds to *K*_2_/*K*_4_ ~ 10^2^. Reducing this anisotropy ratio lowers the required transition field, though it approaches a threshold value. Our investigation of anisotropy variations, both sequentially and independently, revealed the following patterns: reducing only one layer’s anisotropy (lower layer |*K*_4_| = 7 × 10^4^ J/m^3^) permits BP states with marginally decreased field requirements; Bloch points’ formation is suppressed when both confining layers’ anisotropy constants are reduced by an order of magnitude (|*K*_1_,_4_| = 7 × 10^4^ J/m^3^); and increasing the easy-plane magnetic anisotropy constants in either one or both confining layers elevates the magnetic field strength needed to stabilize BP-type states.

The interlayer exchange coupling significantly influences the nucleation field of Bloch points. While all above results assume ferromagnetic interfacial ordering, antiferromagnetic configurations substantially reduce the required nucleation field strength.

### 3.2. Chiral Bobber

Another interesting three-dimensional topological state is the chiral bobber. The observation of Bloch points in our multilayer system naturally suggests the potential formation of chiral bobbers, as both represent three-dimensional topological defects—albeit with distinct structural configurations [35]. While Bloch points manifest as singular, point-like objects with undefined magnetization at their core, bobbers extend this topology by attaching a chiral tail that gradually unwinds into the bulk. The existence of bobbers—a novel type of topological state localized at interfaces and surfaces in chiral magnets—was first predicted theoretically [35]. These structures have now been experimentally confirmed [36]. Chiral bobbers exhibit several remarkable characteristics that distinguish them from other topological textures [7,33,34,35]. They combine features of both 0D (point-like) and 1D (string-like) topological defects; their asymmetric structure features a surface core and bulk tail that respond differently to external stimuli, and the core-tail configuration enables novel responses to both surface and bulk perturbations.

Chiral bobbers typically emerge in systems exhibiting a Dzyaloshinskii–Moriya interaction (DMI); however, their formation is not guaranteed even when DMI is present.

**Figure 3:**
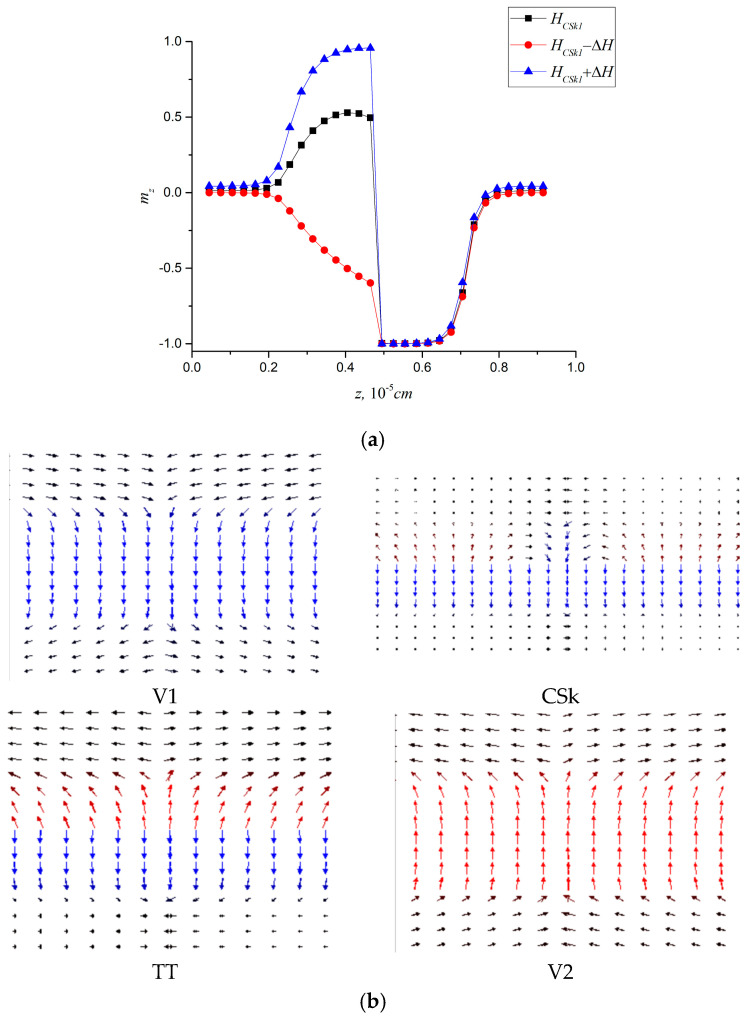
(**a**) Distribution of the *m_z_* component, averaged over the xy plane, plotted as a function of the z coordinate across the film thickness at the parameters, corresponding to the conical skyrmion phase CSk1, (**b**) sequence of magnetic phases emerging during magnetization and reversal processes, *D*_3_ = 2 × 10^−4^ J/m^2^.

In our study, the path to bobber stabilization revealed an intermediate state: conical skyrmions. Under moderate DMI strengths, we initially observed the formation of these hybrid textures, where spins exhibit partial twisting without developing a fully detached bobber tail. We demonstrate that introducing DMI into the inner layer with a lower PMA constant significantly alters the reversal magnetization processes. In contrast to the scenario described in Section 3.1, the magnetization and reversal processes evolve through a different mechanism. Specifically, the Bloch point state becomes replaced by a conical skyrmion state, whose profile is shown in Figure 3a. Figure 3b shows the sequence of phases that emerge during the magnetization reversal process.

**Figure 4:**
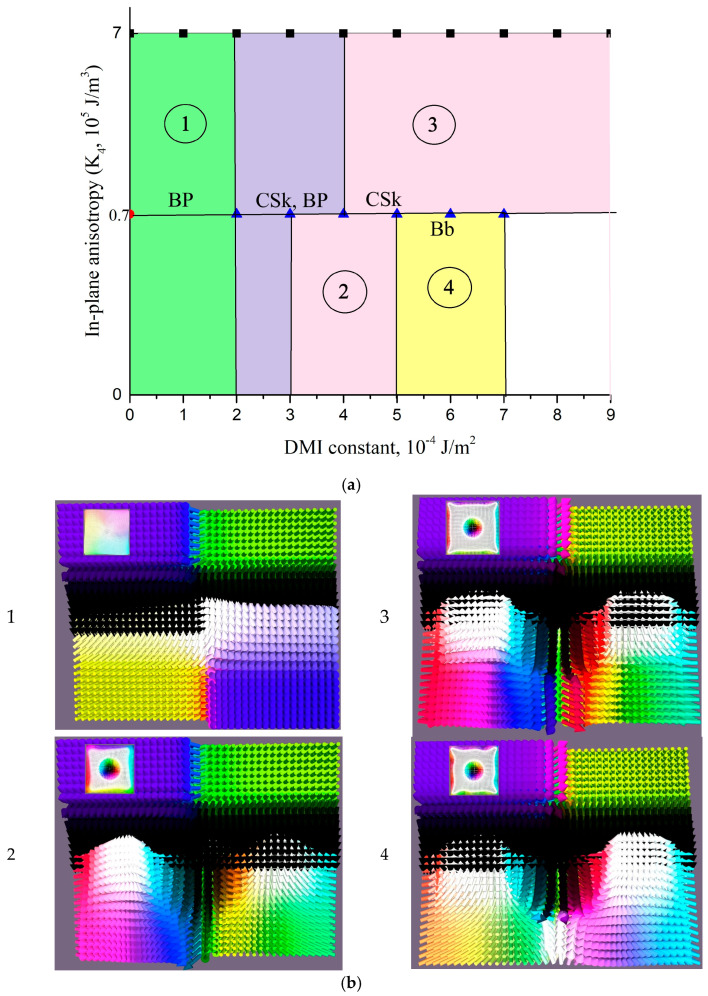
(**a**) Phase diagram illustrating the field-induced topological magnetic states in the four-layer exchange coupled film with an inner chiral layer: BP in green area (1), BP and CSk in purple area, CSk in pink areas (2, 3); Bb in yellow area (4), (**b**) distribution of magnetic moments projected on yz plane in typical 3D structures at following parameters: (1) BP (*D*_3_ = 1 × 10^−4^ J/m^2^, *K*_4_ = 7 × 10^5^ J/m^3^), (2) CSk (*D*_3_ = 4 × 10^−4^ J/m^2^, *K*_4_ = 7 × 10^4^ J/m^3^), (3) CSk (*D*_3_ = 8 × 10^−4^ J/m^2^, *K*_4_ = 7 × 10^5^ J/m^3^), and (4) Bb (*D*_3_ = 7 × 10^−4^ J/m^2^, *K*_4_ = 7 × 10^4^ J/m^3^).

While the Bloch point state still emerges during magnetization at weak DMI strengths, it becomes suppressed as the DMI strength increases. However, increasing the DMI while simultaneously adjusting the easy-plane anisotropy (IMA) of the boundary layer (*K*_4_) enables stabilization of chiral bobber configurations in the system. The complete set of magnetic structures observed in the system is mapped in the phase diagram of Figure 4.

Only through further optimization of DMI strength, layer-specific anisotropy, and interlayer exchange coupling did we achieve the complete transition to well-defined bobber states. This progression—from Bloch points to conical skyrmions to chiral bobbers—highlights the critical role of parameter fine-tuning in stabilizing complex 3D spin textures. Notably, the conical skyrmion phase serves as an essential precursor state that bridges the gap between compact singular defects and extended chiral structures.

### 3.3. Skyrmion Tubes

Let us consider another topological defect that is realized in the film under consideration. These skyrmion tubes can be considered as a further extension of 3D skyrmions, which can be realized in the system under consideration. Skyrmion tubes represent a natural three-dimensional extension of magnetic skyrmions, where the 2D whirl-like spin texture is uniformly continued along the third spatial dimension, forming a cylindrical, vortex-like structure [32,37,38,39,40,41,42,43]. These tubes retain the topological protection of their 2D counterparts while exhibiting unique behaviors in bulk materials and thin films. Skyrmion tubes are characterized by a non-trivial winding number (topological charge ±1) that persists along their length. Their stability arises from interactions like the Dzyaloshinskii–Moriya interaction (DMI) or dipole–dipole coupling, depending on the material. Skyrmion tubes can terminate at Bloch points—singularities where the magnetization becomes undefined. These points act as topological “sources” or “sinks” for the tubes. When skyrmion tubes bend and close into a torus, they form hopfions.

In our thin film system, skyrmion tubes emerge during the magnetization process when the DMI is present in two layers. The magnetization and reversal mechanisms exhibit analogous to previously studied systems, characterized by the nucleation of 3D topological states. As observed in single chiral layer systems, weak DMI results in localized chiral textures that condense into a conical skyrmion (CSk) state as the field increases. This CSk state then evolves into a Bloch point (BP) during magnetization or tail-to-tail (TT) state during the reversal process. However, elevated DMI strength alongside diminished in-plane anisotropy eliminates BP-like structures, unlocking new transformation pathways.

**Figure 5:**
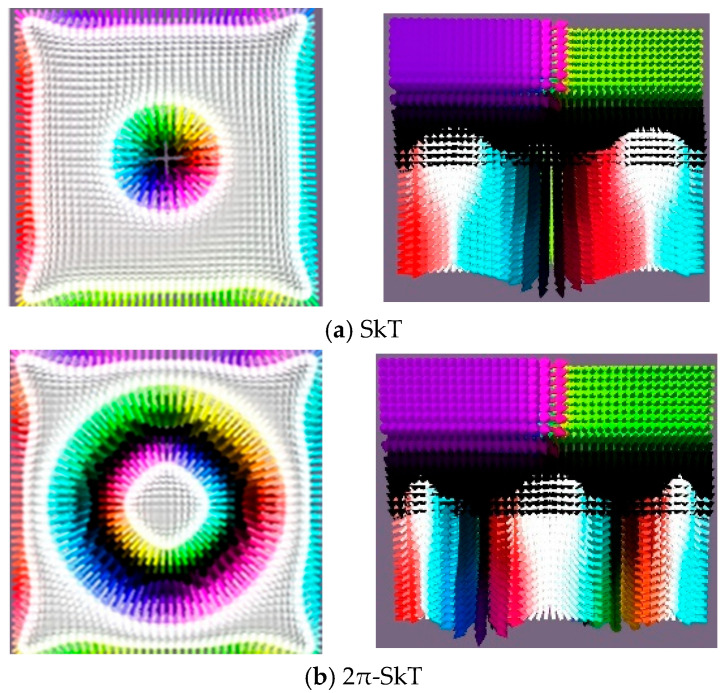

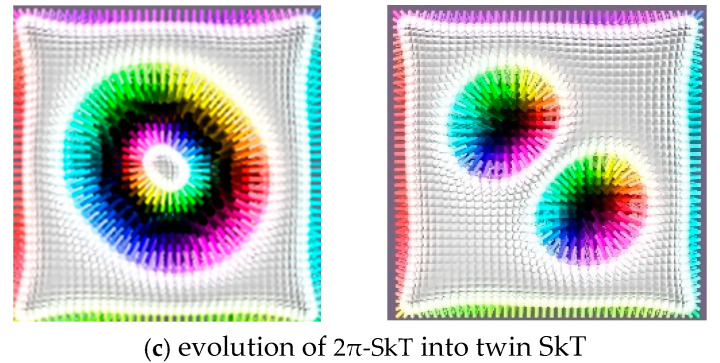
Skyrmion tubes induced by magnetic field ***H*** in the film at the following parameters (**a**) SkT at *D* = 6 × 10^−4^ J/m^2^, *H_SkT_* = 720 kA/m, (**b**) 2πS-kT at *D* = 8 × 10^−4^ J/m^2^, *H*_2πSkT_ = 840 kA/m; and (**c**) Twin SkT, *D* = 7 × 10^−4^ J/m^2^, *H_TSk_* = 720 kA/m, *K*_4_ = 7 × 10^4^ J/m^3^.

The magnetization and reversal mechanisms follow behavior that is analogous to previously studied systems, involving the formation of topological defects. As observed in single chiral layer systems, a weak DMI leads to localized chiral structures in the chiral layers that collapse into a conical skyrmion (CSk) state as the field increases. This CSk state then evolves into either a Bloch point (BP) or tail-to-tail (TT) state. However, strong DMI combined with reduced in-plane anisotropy suppresses BP states and enables alternative phase transition pathways. In systems with two chiral layers (*D*_3_ = *D*_4_ = *D*), we observe similar phase transitions, with the key distinction that a conical skyrmion transforms into one of several configurations, depending on the parameter ratios: a skyrmion tube (SkT), a 2π-skyrmion tube (2π-SkT), or a pair of skyrmion tubes (twin SkT), shown in Figure 5 in the planar (x-y) and the depth-resolved (x-z) projections.

**Figure 6:**
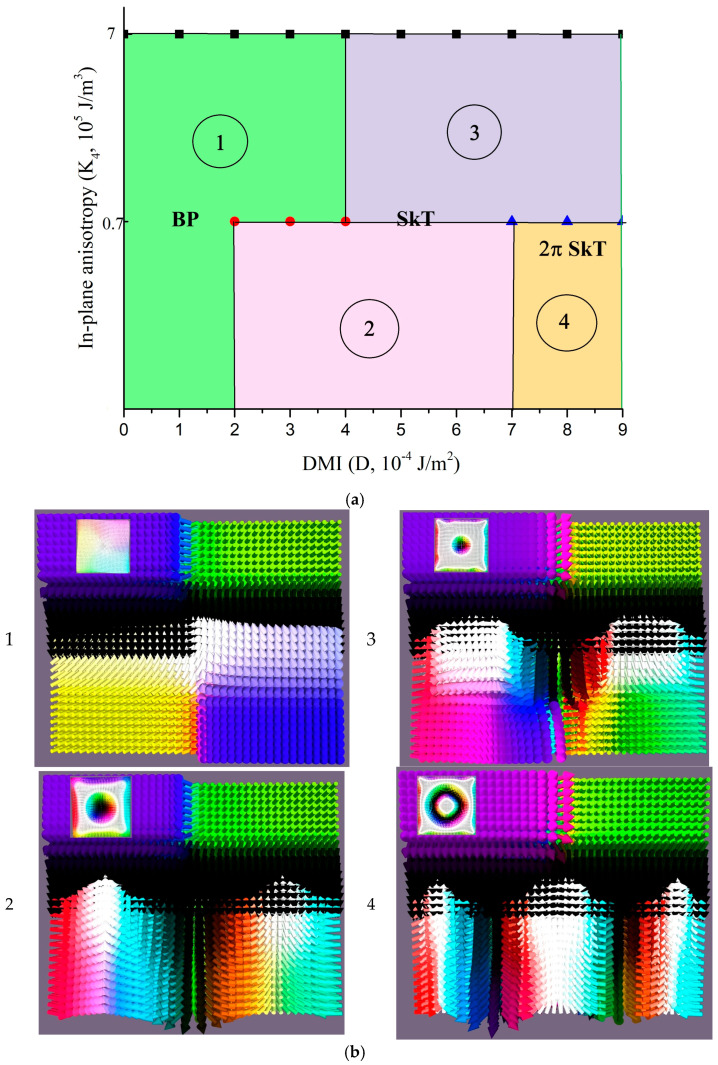
(**a**) Phase diagram of field-induced topological magnetic states in a four-layer exchange-coupled film with adjacent chiral layers (*D*_3_ = *D*_4_), showing the stabilization regions for Bloch-point-like states (BP)—green area (1), skyrmion tubes (SkT)—pink and purple areas (2, 3), and 2π-skyrmion tubes (2π-SkT)—yellow area (4). (**b**) Representative visualizations of the topological structures, corresponding to specific points within the regions of the phase diagram defined by parameters (*D*, *K*_4_).

The complete phase diagram, mapping the stability regions of these states against the system parameters, is presented in Figure 6. As seen here, the system exhibits several types of 3D skyrmions: skyrmion tubes, and 2π-skyrmion tubes that decay into two individual tubes with increasing DMI strength.

Thus, we demonstrate that nanoscale films supporting Bloch point states serve as a remarkable platform for generating diverse topological defects. Notably, while our current study focuses on the observed defect states, we anticipate that hopfion configurations—arising from the bending of skyrmion tubes—may also emerge in this system. However, a detailed investigation of such hopfion states lies beyond the scope of this work and will be addressed in future studies.

## 4. Conclusions

In this study, we investigated the stabilization and control of three-dimensional topological magnetic defects—including Bloch points, chiral bobbers, and skyrmion tubes—in exchange-coupled ferromagnetic multilayers. We have demonstrated that the formation and stability of these exotic spin textures are governed by the interplay between the Dzyaloshinskii–Moriya interaction (DMI), magnetic anisotropy, and interlayer exchange coupling. The competition between PMA and IMA layers, coupled with tunable DMI strengths, enables selective nucleation of distinct topological states. Bloch points emerge in systems with weak or absent DMI, while chiral bobbers and skyrmion tubes dominate at higher DMI values, with critical thresholds identified for phase transitions. Layer-specific anisotropy gradients act as a powerful tool to manipulate defect morphology. For instance, reducing easy-plane anisotropy in boundary layers suppresses Bloch points, whereas optimized PMA/IMA ratios stabilize hybrid configurations like conical skyrmions. Our findings, supported by micromagnetic simulations, reveal the critical role of DMI and layer anisotropy in governing the formation and stability of these topological states. The novelty of our approach lies in the ability to generate and manipulate multiple topological states within a single system (artificially layered magnetic structure) by tuning the layer properties, such as anisotropy and interlayer exchange coupling.

This multilayer platform offers decisive advantages over conventional heavy-metal/ferromagnet (HM/FM) bilayers for device integration. Firstly, it can solve the critical challenge of weak electrical readout signals. Three-dimensional states like skyrmion tubes, coherently stabilized across multiple ferromagnetic layers, generate a topological Hall or magnetoresistance signal that is multiplicatively enhanced, improving the signal-to-noise ratio. Secondly, it overcomes the inherent trade-off between skyrmion thermal stability and mobility. Our architecture allows for the independent optimization of these properties across different layers; for example, deeper layers can be engineered for strong pinning (high anisotropy) while surface layers are tailored for efficient current-driven motion (lower anisotropy, higher spin–orbit torque efficiency). This decoupling of functionality, impossible in a single interface, enables the custom design of topological states with bespoke properties. Finally, for neuromorphic computing, the controlled transitions between different topological states introduce robust multi-state functionality within a single device element, which is ideal for representing multi-level synaptic weights.

Therefore, transitioning from conventional bilayers to engineered multilayers is a necessary step, moving from working with fixed interfacial properties to actively designing a three-dimensional magnetic energy landscape that overcomes the fundamental limitations of signal strength, stability, and functional complexity for real-world spintronic and neuromorphic devices.

## Data Availability

Data are contained within the article.
